# Effects of fulvic acid addition on laying performance, biochemical indices, and gut microbiota of aged hens

**DOI:** 10.3389/fvets.2022.953564

**Published:** 2022-09-02

**Authors:** Gengsheng Xiao, Shun Liu, Xia Yan, Yang Yang, Qien Qi, Xin Feng, Li Gong, Huihua Zhang

**Affiliations:** ^1^School of Life Science and Engineering, Foshan University, Foshan, China; ^2^State Key Laboratory of Livestock and Poultry Breeding, Institute of Animal Science, Guangdong Academy of Agricultural Sciences, Guangzhou, China

**Keywords:** fulvic acid, Dawu Golden Phoenix laying hen, production, egg quality, microbiota

## Abstract

The purpose of this study was to appraise the effect of fulvic acid on production, biochemical indices, and gut microbiota of laying hens. A total of 252 Dawu Golden Phoenix laying hens (55-week-old) were allotted to two treatments randomly, each with six replicates and 21 hens per replicate, including the control group (CG) and fulvic acid (500 mg/kg) group (FA). The trial period was 8 weeks. Adding FA raised egg weight (*P* = 0.03), shell-breaking strength (*P* = 0.03), and reduced egg breaking rate (*P* < 0.01), compared with CG. There was no difference in eggshell thickness and egg shape index between the two treatments; however, the FA group increased egg production by 1.45% and reduced the feed-to-egg ratio by 0.09. Moreover, dietary FA decreased the aspartate aminotransferase levels in serum (*P* = 0.04), and glutathione peroxidase and total antioxidant capacity were increased (*P* = 0.02 and 0.04, respectively). Despite this, the two groups had no differences in the alpha diversity indices (PD_whole trees, Shannon, Ace, Simpson, Chao1, and goods_coverage). Obviously, at the phylum level, the abundances of *Firmicutes* were improved (*P* < 0.01), *Actinobacteriota* (*P* < 0.01), and *Proteobacteria* (*P* < 0.01) were reduced by dietary FA. Supplementation with FA could improve the abundances of *Megamonas* (*P* < 0.01) and reduce *Enterobacter* (*P* < 0.01) at the genus level. To sum up, this study showed the addition of 500 mg/kg FA may boost production and egg quality and modulate the cecal microflora abundance and serum biochemical indices of laying hens.

## Introduction

In the late laying stage (after 40 weeks), the production and eggshell quality of laying hens reduced as they became older (1). Furthermore, thinner shells, higher breakage rates, and inferior egg quality have all been linked to ill-health among older hens (2).

Fulvic acid (FA) is an organic compound extracted from peat and various forms of coal, such as lignite and worn coal, which is created as a result of the breakdown of microbial matter, animal and plant leftovers, as well as the buildup of processes (3). FA is a low-molecular-weight molecule with high biological activity dissolved in alkali and acid liquids (4). In addition, FA has been shown to be compatible with all aquatic creatures (5). Numerous reactive functional groups are included in fulvic acids, such as hydroxyl, quinone, phenol, and carboxyl groups (6), which confer the following beneficial activities on FA: antioxidant activity (7), antiviral and anti-inflammatory effects (8), stimulation of immunity (9), and metal chelation (10). Mao (11) appraised dietary fulvic acid on production, oxidative, and immunological parameters in broilers and observed that FA supplementation resulted in higher weight gain in broilers than controls. Semjon et al. (12) studied the humic compounds on meat quality and found that dietary 1% humic can significantly reduce broiler chicken thigh meat's cooking water loss rate. Moreover, FA could improve production and meat quality in growing-finishing pigs (4). Chang et al. (9) confirmed that FA positively affects growth performance and immunity in growing pigs. However, there are still few studies about the effects of FA on performance, serum biochemical indexes, and gut microbes of aged hens. This work aimed to investigate the effects of dietary FA on production, biochemical indicators, and gut microflora at the late stage of hens.

## Materials and methods

### Birds, diets, and experimental design

A total of 252 Da Wu Golden Phoenix egg-laying hens, at 55 weeks of age were, randomly placed into two treatments of six replicates each, with 21 hens per treatment. The control group was fed a basal diet, and the fulvic acid group was fed a basal diet supplemented with 500 mg/kg of FA purchased from Beijing Sloan Biotechnology Co., Ltd. The study began on the 55th week and continued for 8 weeks. The basal diet was created according to the nutritional needs of laying hens (2012), and the feed components and nutrient compositions are shown in Table 1. The hens were enclosed in cages that resembled the letter A when viewed from the side and shared a room that was cleaned and disinfected daily at 26°C, 60–65% humidity and a light cycle for 16 h. 100 g of feed every day at 6 a.m. and 1 p.m., and collect eggs every day at 6 p.m. All hens were given fresh water, fed, eggs collected and weighed daily.

**Table 1:** Ingredients in the basic diets and nutritional composition.

**Feed ingredients**	**%**	**Nutrient composition**	**%**
Corn	60.80	ME (kcal/kg)	4,041.6
Soybean meal	26.00	CP	17.00
Limestone	7.74	Calcium	3.25
Soybean oil	2.62	Phosphorus	0.50
Calcium bicarbonate	1.40	Salt	0.03
Lysine	0.18	Lysine	0.998
DL-Methionine	0.18	DL-Methionine	0.435
Threonine	0.08		
1% premix	1.00		
Total	100		

1% premix includes the following nutrients: vitamin A, 12,500 IU; vitamin D3, 4,500 IU; vitamin E, 25 mg; vitamin B, 2 mg; vitamin K, 3 mg; vitamin B2, 30 mg; vitamin B12, 1 mg; niacin 3 g; choline, 1,500 mg; pantothenic acid 700 mg; folic acid, 600 mg; Fe, 9 mg; Cu, 9 mg; Mn, 9 mg; I, 43 mg; Se, 30 mg; biotin, 0.2 mg.

### Productive performance and egg quality

During the 55–62 week period, feed intake, egg number, and egg breaking were all documented daily. The number of broken eggs was used to compute the egg breaking rate. The average egg weight, feed/egg, and egg production rate were calculated weekly. During the last week of the test, 36 eggs were randomly chosen from each group (six eggs per replication). The 72 eggs were tested for shell-breaking strength (Egg Force Reader, Orka Food Technology Ltd), Haugh units (Egg Analyzer, Orka Food Technology Ltd, Israel), and shell thickness (Eggshell Thickness Gauge, Orka Food Technology Ltd). The egg shape index was calculated using the width-length ratio (%).

### Serum biochemical indices and antioxidant parameters

In the final experiment, every replicate had one bird randomly selected; blood samples were obtained after a 12 h meal fast. Pterygoid vein blood samples were collected, and serum was centrifuged at 3,000 × g for 15 min at 4°C after 2 h of quiescence. We used Nanjing Jiancheng Bioengineering Institute (Nanjing, Jiangsu, China) kits to determine serum triglycerides, high-density lipoprotein, total cholesterol, low-density lipoprotein, aspartate aminotransferase (AST), total bilirubin, and alanine aminotransferase (ALT). Parameters related to antioxidants included glutathione peroxidase (GSH-Px), malondialdehyde (MDA), full antioxidant capacity (T-AOC), and total superoxide dismutase (T-SOD) and were also examined according to the kits (Nanjing, Jiangsu, China). Following the blood collection, the hens were slaughtered and bled *via* cervical dislocation. Aseptically collected left and right cecum of each chicken was placed in sealed vials and frozen in liquid nitrogen within minutes. After that, the specimens were stored at −80°C to make further analyses possible.

### Cecal digesta DNA extraction and 16S rRNA sequencing analysis

After the investigation, the cecal microbiota of the six chosen chickens per group was assessed. After removing the cecal contents aseptically, the digest was snap-dried in liquid nitrogen, cooled to−80°C, and total genome DNA was extracted using the cetyltrimethylammonium bromide technique. DNA content and purity were determined by 1.5% agarose gel electrophoresis. Amplification of the V3/V4 region of the 16S rRNA gene was achieved by using primers 341F (5'-CCTACGGGNGGCWGCAG-3') and 804R (5'-GACTACHVGGGTATCTAATCC-3') (13). Fast Length Adjustment of Short Reads (V1.2.7) was used to merge paired-end reads into clean reads of high quality, and QIIME (Quantitative Insight into Microbial Ecology) was used in a quality control pipeline to filter the raw sequences and identify quality segments (14). Clusters of high-quality sequences have been categorized as operational taxonomic units (OTUs) based on a similarity of 97 percent, and sample OTU sequences have been categorized as taxa using the SILVA database (15). Two groups were visualized using a Venn diagram to show shared and distinct OTUs. The alpha diversity was calculated with the vegan package. For beta-diversity analysis, a principal coordinate analysis (PCoA) based on Bray-Curtis distance was used. STAMP with *t*-tests was used to examine the differences in microbial abundances (16).

### Statistical analysis

All data were expressed as means ± SD, using an independent samples *t*-test with SPSS 25.0 software (SPSS, Inc., Chicago, IL), including fixed effects treatments in the model, and presented using GraphPad Prism version 9 (GraphPad Software, La Jolla, CA). A *P*-value < 0.05 was considered significant (*P* < 0.05, *P* < 0.01), while 0.05 < *P*-value < 0.10 was considered tendencies.

## Results

### Production and egg quality

In this work, compared with the control group, shell-breaking strength (*P* = 0.03) and egg weight (*P* = 0.03) were improved by FA (Table 2). The egg breaking rate of FA was reduced by 0.23% (*P* < 0.01; Table 2). However, eggshell thickness and the egg shape index between the two treatments were no different, but the FA group increased egg production by 1.45% and reduced the feed-to-egg ratio by 0.09.

**Table 2:** Egg productive performance and quality.

**Items**	**CG**	**FA**	**SEM**	***P*** **-value**
**Productive performance**				
Egg production rate, %	81.92 ± 5.28	83.37 ± 4.38	1.21	0.59
Egg weight, g	60.79 ± 0.29	61.08 ± 0.16	0.06	0.03
Feed-to-egg ratio, g:g	2.03 ± 0.14	1.94 ± 0.11	0.32	0.27
Egg breaking rate, %	0.65 ± 0.15	0.42 ± 0.12	0.35	<0.01
**Egg quality**				
Egg shape index	1.31 ± 0.01	1.30 ± 0.02	0.01	0.16
Shell breaking strength	3.81 ± 0.08	3.92 ± 0.09	0.03	0.03
kg/cm^2^				
Shell thickness, mm	0.38 ± 0.02	0.39 ± 0.02	0.01	0.20
Haugh units	71.83 ± 7.34	77.60 ± 4.26	1.73	0.13

CG, control group; FA, fulvic acid.

### Blood biochemical parameters

The AST of the treatment group was lower than that of the control group (*P* = 0.04; Table 3). Compared with the control group, the total bilirubin in the FA tended to decrease (*P* = 0.06; Table 3). The T-AOC and GSH-Px of the FA were increased (*P* < 0.05; Table 3).

**Table 3:** Serum biochemical indices and antioxidant parameters.

**Items**	**CG**	**FA**	**SEM**	***P*** **-value**
**Serum biochemical indices**				
Triglycerides, mmol/L	2.42 ± 0.64	3.49 ± 0.54	0.17	0.22
Total bilirubin, μmol/L	112.21 ± 14.5	82.66 ± 13.5	4.05	0.06
High-density lipoprotein, mmol/L	5.18 ± 0.86	5.67 ± 0.78	0.24	0.33
Low-density lipoprotein, mmol/L	0.32 ± 0.10	0.26 ± 0.09	0.28	0.28
Total cholesterol, mmol/L	3.30 ± 1.06	4.85 ± 0.66	0.25	0.30
AST, U/L	28.27 ± 6.68	19.80 ± 5.25	1.74	0.04
ALT, U/L	12.85 ± 3.25	10.48 ± 2.65	0.86	0.20
**Antioxidant parameters**				
T-AOC, U/ml	6.67 ± 1.68	4.40 ± 1.75	0.50	0.04
MDA, nmol/ml	5.65 ± 1.38	5.83 ± 1.65	0.41	0.70
GSH-Px, U/ml	594.36 ± 56.67	698.34 ± 71.34	18.59	0.02
T-SOD, U/ml	73.38 ±7.42	82.97 ± 3.41	1.67	0.12

CG, control group; FA, fulvic acid; T-AOC, total antioxidant capacity; MDA, malondialdehyde; GSH-Px, glutathione peroxidase; T-SOD, total superoxide dismutase.

### Cecal contents microbial

**Figure 1:**
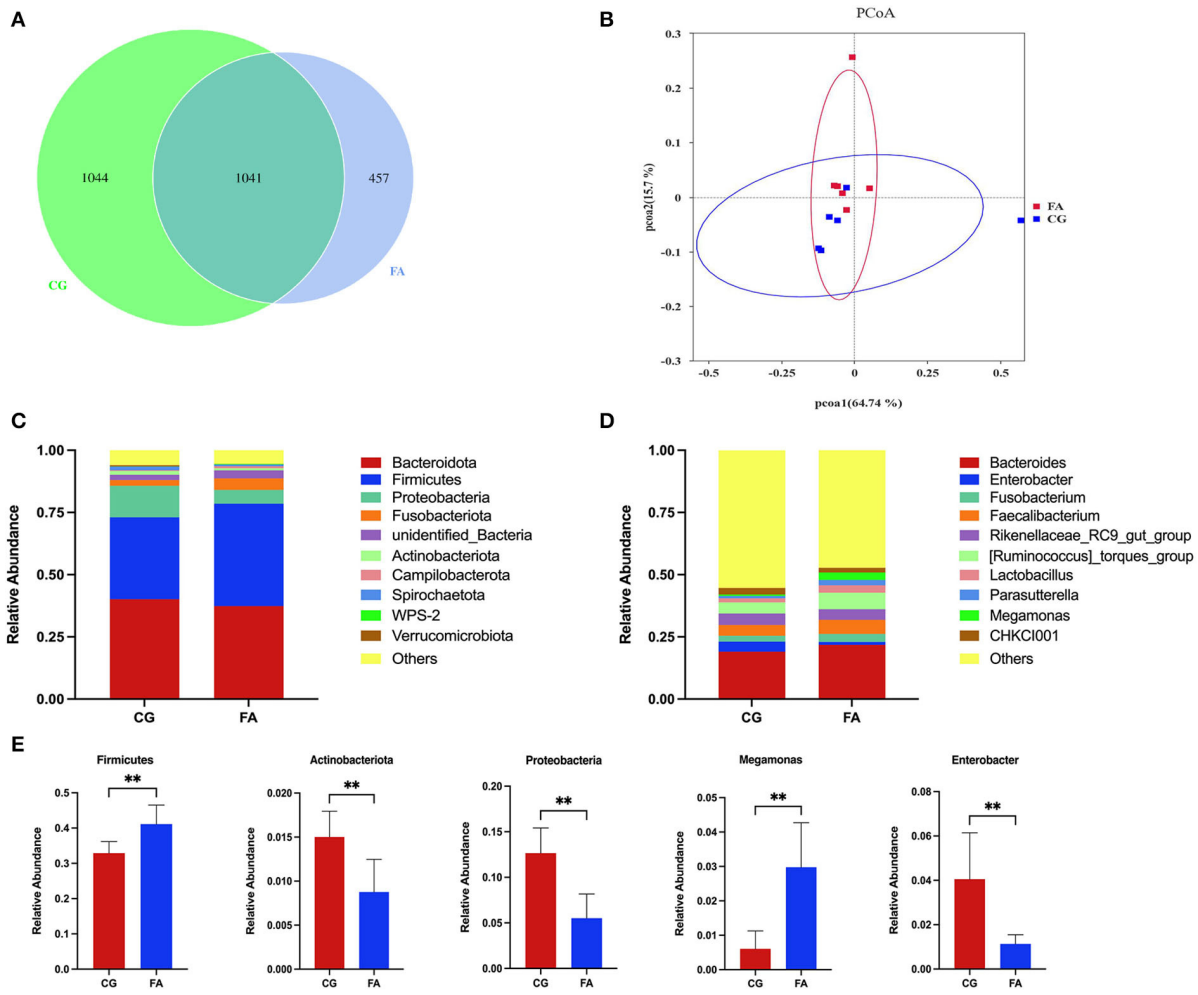
Cecal contents microbial composition. **(A)** Venn diagram of OTUs number; **(B)** PCoA of the cecal microbiota; **(C)** Phylum-level taxonomic composition of the cecal microbiota; **(D)** Genus-level taxonomic composition of the cecal microbiota. **(E)** Relative abundance of *Firmicutes, Actinobacteriota, Proteobacteria, Megamonas*, and *Enterobacter*. CG, control group; FA, fulvic acid. Significant deviations are denoted by asterisks (**P < 0.01).

The two groups received 2,542 OTUs, with 457 and 1,044 OTUs found only in the FA and control groups, respectively (Figure 1A). FA supplementation did not affect the alpha diversity indices (Chao1, Shannon, Ace, Simpson, PD_whole trees, and goods_coverage) (Table 4). PCoA analysis was performed using the Bray-Curtis similarity technique. The first principal component (PCoA1) and second (PCoA2) explained 64.74 and 15.7% of the variation in microbial diversity, respectively (Figure 1B). The samples in the control and FA groups were tightly packed and not far apart, as shown in the primary coordinate analysis diagram. According to the taxonomic study, the structure of the cecal flora did not alter following FA therapy. Phylum-wise, *Bacteroidetes* (> 37%) and *Firmicutes* (>32%) rank first and second, respectively (Figure 1C). Compared with the control group, *Firmicutes* were elevated by FA (*P* < 0.01; Figure 1E). There was a reduction in *Actinobacteriota* and *Proteobacteria* in the FA group (*P* < 0.01; Figure 1E). In addition, *Bacteroidota* dominates (>19%), followed by *Ruminococcus_torques_group* (>4%), *Faecalibacterium* (>4%), and *Rikenellaceae_RC9_gut_group* (>4%); the rest of the genera listed were all below 2% at the genus level (Figure 1D). Supplementation with FA at genus levels elevated *Megamonas* and reduced *Enterobacter* (*P* < 0.01; Figure 1E).

**Table 4:** Alpha diversity indexes of cecal microbiota.

**Items**	**CG**	**FA**	**SEM**	***P*** **-value**
Ace	1012.25 ± 129.18	961.09 ± 80.34	31.05	0.43
Chao1	994.57 ± 115.81	956.56 ± 89.28	29.85	0.54
Shannon	6.84 ± 0.50	6.72 ± 0.25	0.11	0.61
Simpson	0.97 ± 0.03	0.97 ± 0.01	0.01	0.74
Goods_coverage	1.00 ± 0.01	1.00 ± 0.01	0.01	0.23
PD_whole tree	65.80 ± 10.09	60.06 ± 5.70	2.37	0.25

CG, control group; FA, fulvic acid.

## Discussions

Dawu Golden Phoenix laying hens will be culled after 72 weeks when the egg production rate is below 80% (17). In the layer industry, the most pressing economic issues are production and egg quality. Shell thickness and shell strength are essential egg qualities. Eggshell strength affects egg breaking rate and compression resistance, making the eggs convenient to transport and store over long distances. An et al. (18) test proved that adding 0.5 g/kg fulvic acid to the feed of laying hens can increase the egg production rate and average egg weight by 5.20 and 7.40%, respectively, and reduce the feed-to-egg ratio, broken egg rate, and total egg weight. Wang et al. (19) found that adding 0.048% fulvic acid could increase the activity of plasma superoxide dismutase in 42-day-old broilers and reduce the content of malondialdehyde. In addition, Bi (20) administered 5 g/kg fulvic acid and sodium fulvic acid to mice to investigate the acute toxicity of both, and none of the experimental mice died, indicating that the oral toxicity of fulvic acid is extremely low. This explains why the fulvic acid dose chosen for this study was 500 mg/kg. In this work, the addition of FA increased the egg production rate by 1.45%, while the feed-to-egg ratio decreased by 0.09, compared with the control group, but there was no statistically significant difference. Hudák et al. (21) showed that dietary supplementation with natural and acidified humus did not improve broiler body weight and feed conversion ratio. Interestingly, Domínguez-Negrete et al. (22) found different dietary doses of humic acid did not significantly change the daily gain and feed conversion ratio. In the Prokešová et al. (23) study, the humic substances (HS) incorporated into the diet (HS0–6) did not affect the production of Clarias gariepinus. However, broilers fed with FA gained significantly more body weight than those fed with control diets, and their FCR was also lower than those fed with control diets (11). Few studies have been conducted on fulvic acid in late-laying hens. The egg production for humate and probiotic-fed hens was not different, but both groups produced more eggs than the control-fed hens (24). Our study observed increased egg weight and eggshell strength with dietary FA. Nevertheless, there is still relatively low knowledge of different FA sources and products. Dosage, synthetic form of FA, and animal species may also contribute to inconsistent results.

The existence of serum enzymes and their levels in the blood can help determine the extent of organ or tissue damage. Because they are synthesized in the liver, ALT, AST, and total bilirubin are essential indices for monitoring the liver function of chickens. Diets containing potassium humate had lower AST values than diets containing 17.5% canola meal (25). In this experiment, we found that fulvic acid was able to reduce serum AST levels and tended to decrease total bilirubin. This may indicate that humic acid can protect the liver by reducing free radicals produced during liver metabolism (26). Nevertheless, in Prokešová et al. (23) study, the activity of ALT, AST, and lactate dehydrogenase enzymes were within the species-specific optimal physiological range, and no differences were observed between the Clarias gariepinus tested groups after 28 and 56 days of HS feeding. Different results may be attributed to animal species, dosage, synthetic form, etc.

Antioxidant mechanisms in animals are essential for their health, growth, productivity, and economic rewards. In organisms, GSH-Px and T-SOD are vital antioxidant enzymes in which superoxide radicals and peroxides are scavenged, and the generation of hydroxyl radicals is reduced. A measure of lipid peroxidation mediated by oxygen free radicals is MDA content, and T-AOC measures the body's antioxidant capacity. The broilers fed FA-containing diets were more active at T-SOD and GSH-Px, and their MDA levels were lower than those provided in control diets, but when administered at a high level of FA (1 g kg^−1^), T-SOD and GSH-Px activities did not increase further, and the MDA level decreased in comparison to the moderate group (0.5 g kg^−1^) (11). High doses of fulvic acid may not necessarily benefit the animal body. Dietary FA improved the activities of GSH-Px and T-SOD in *L. vannamei* (27). In this study, FA increased the serum level of T-AOC and GSH-Px, suggesting FA may be able to protect laying hens against oxidation damage more effectively. It could be attributed to the antioxidant properties of FA (7).

There is mounting evidence suggesting that feed supplementation affects gut microbiota regulation and, thus, animal performance (28). Recent studies have indicated the intestinal flora acts as the main barrier against infection and colonization by pathogenic bacteria, implying the importance of its role in the treatment and prevention of diseases (29). Studies on the effects of FA supplementation on laying hens' cecal microbiota are limited. Our study showed that intestinal microflora diversity was not significantly different between the FA and control groups. Interestingly, dietary FA could increase the relative abundance of *Firmicutes* and *Megamonas* and reduce *Proteobacteria, Actinobacteriota*, and *Enterobacter*. *Firmicutes* facilitate cellulose digestion in the gut, so their higher abundance during growth and development aids in meeting animals' nutritional and energetic needs (30). Furthermore, *Firmicutes* is composed of a variety of gram-positive bacteria, some of which help prevent pathogen intrusion and balance microflora in the intestine (31). In addition, *Actinobacteria* can easily transfer synergy between themselves and a partner or host into pathogenic interactions (32). In diarrheal goats, the relative abundance of Actinobacteria was dramatically increased (33). As a group, *proteobacteria* are primarily composed of gram-negative bacteria, such as *Escherichia coli, Salmonella, Helicobacter pylori*, and *Vibrio cholerae*. Animals could suffer from diarrhea, gastritis, vomiting, gastrointestinal ulcers, and even death from the bacteria, posing a serious health threat (34). *Megamonas* is a prominent member of the *Veillonellaceae* family. This genus produces acetic and propionic acids using fermentable fibers as their substrate (35). Infections of the urinary tract, lower respiratory tract, bloodstream, and damage to soft tissues are caused by *Enterobacter* (36). Therefore, in this experiment, FA supplementation increased the abundance of beneficial bacteria and reduced the abundance of harmful bacteria in the gut. FA has good anti-inflammatory, hemostasis, antiviral, and other effects. As a feed additive, it can improve the immunity of chickens without toxic or side effects. It should be noted that FA can improve the production performance and egg quality of laying hens in various ways, but it may be mainly achieved by regulating the body's antioxidant capacity and intestinal flora. However, the specific impact mechanism still needs to be further explored. Further research needs to be conducted on the role of fulvic acid in eggshell calcium deposition.

## Conclusions

According to this study, the addition of 500 mg/kg of fulvic acid enhanced eggshell quality, egg weight, and serum antioxidant parameters and reduced the egg-breaking rate in hens. Although fulvic acid supplementation had no influence on the alpha diversity index in the cecal microbiota, it did increase the relative abundance of *Firmicutes* and *Megamonas* while decreasing the *Actinobacteriota, Enterobacter*, and *Proteobacteria*. The findings shed light on how FA can be utilized to improve the laying hen's production cycle and egg quality, which has significant implications for the industry's long-term health. However, more research on the mechanism and ideal FA ratio is required.

## Data availability statement

The datasets presented in this study can be found in online repositories. The names of the repository/repositories and accession number(s) can be found below: https://www.ncbi.nlm.nih.gov/, BioProject PRJNA838954.

## Ethics statement

The animal study was reviewed and approved by Ethical Committee and conducted under the supervision of the Institutional Animal Care and Use Committee of Foshan University (Foshan, China).

## Author contributions

HZ and LG designed the experiment. GX and SL finished the statistical analysis of all data and the original draft written. GX, SL, and YY conducted the animal feeding. XY and QQ participated in the sample collection. All authors read and approved the final manuscript.

## Funding

The work was supported by the Guangdong Province Modern Agriculture Poultry Industry technology system innovation team construction project (2021KJ128) and the Discipline Construction Program of Foshan University (CGZ0400162).

## Conflict of interest

The authors declare that the research was conducted in the absence of any commercial or financial relationships that could be construed as a potential conflict of interest.

## Publisher's note

All claims expressed in this article are solely those of the authors and do not necessarily represent those of their affiliated organizations, or those of the publisher, the editors and the reviewers. Any product that may be evaluated in this article, or claim that may be made by its manufacturer, is not guaranteed or endorsed by the publisher.

## References

[1] Bain MM Nys Y Dunn IC. Increasing persistency in lay and stabilising egg quality in longer laying cycles. what are the challenges? Br Poult Sci. (2016) 57:330–8. 10.1080/00071668.2016.1161727 26982003 PMC4940894

[2] Gan L Zhao Y Mahmood T Guo Y. Effects of dietary vitamins supplementation level on the production performance and intestinal microbiota of aged laying hens. Poult Sci. (2020) 99:3594–605. 10.1016/j.psj.2020.04.007 32616256 PMC7597815

[3] Janoš P. Separation methods in the chemistry of humic substances. J Chromatograph A. (2003) 983:1–18. 10.1016/S0021-9673(02)01687-4 12568366

[4] Bai H Chang Q Shi B Shan A. Effects of fulvic acid on growth performance and meat quality in growing-finishing pigs. Livest Sci. (2013) 158:118–23. 10.1016/j.livsci.2013.10.013

[5] Islam KMS Schuhmacher A Gropp J. Humic acid substances in animal agriculture. Pak J Nutr. (2005) 4:126–34. 10.3923/pjn.2005.126.134

[6] MacCarthy P. The principles of humic substances. Soil Sci. (2001) 166:738–51. 10.1097/00010694-200111000-00003

[7] Rodríguez NC Urrutia EC Gertrudis BH Chaverri JP Mejía GB. Antioxidant activity of fulvic acid: a living matter-derived bioactive compound. J Food Agricult Environ. (2011) 9:123–7.

[8] Yang HL Chiu HC Lu FJ. Effects of humic acid on the viability and coagulant properties of human umbilical vein endothelial cells. Am J Hematol. (1996) 51:200–6. 10.1002/(SICI)1096-8652(199603)51:3<200::AID-AJH4>3.0.CO;2-0 8619400

[9] Chang Q Bai H Shi B Shan A Wei C Yu C. Effects of dietary FA on the growth performance, serum biochemical indices, routine blood parameter and immunity of growing swine. Chin J Anim Nutr. (2013) 25:1836–42.

[10] Plaza C García-Gil JC Polo A Senesi N Brunetti G. Proton binding by humic and fulvic acids from pig slurry and amended soils. J Environ Qual. (2005) 34:1131–7. 10.2134/jeq2004.0378 15888899

[11] Mao Y. Modulation of the growth performance, meat composition, oxidative status, and immunity of broilers by dietary fulvic acids. Poult Sci. (2019) 98:4509–13. 10.3382/ps/pez281 31115462

[12] Semjon B Marcincakova D Korenekova B Bartkovsky M Nagy J Turek P. Multiple factorial analysis of physicochemical and organoleptic properties of breast and thigh meat of broilers fed a diet supplemented with humic substances. Poult Sci. (2020) 99:1750–60. 10.1016/j.psj.2019.11.012 32111335 PMC7587867

[13] Gong L Xiao G Zheng L Yan X Qi Q Zhu C. Effects of dietary tributyrin on growth performance, biochemical indices, and intestinal microbiota of yellow-feathered broilers. Animals. (2021) 11:3425. 10.3390/ani11123425 34944202 PMC8697914

[14] Caporaso JG Kuczynski J Stombaugh J Bittinger K Bushman FD Costello EK. QIIME allows analysis of high-throughput community sequencing data. Nat Methods. (2010) 7:335–6. 10.1038/nmeth.f.303 20383131 PMC3156573

[15] Quast C Pruesse E Yilmaz P Gerken J Schweer T Yarza P. The SILVA ribosomal RNA gene database project: improved data processing and web-based tools. Nucleic Acids Res. (2013) 41:D590–6. 10.1093/nar/gks1219 23193283 PMC3531112

[16] Parks DH Tyson GW Hugenholtz P Beiko RG. STAMP: statistical analysis of taxonomic and functional profiles. Bioinformatics. (2014) 30:3123–4. 10.1093/bioinformatics/btu494 25061070 PMC4609014

[17] Li H. High-Yielding Red-Feather Pink-Shell Laying Hens—Dawu Golden Phoenix. Hebei Agriculture (2021).

[18] An X Qi J Tong B Luo X Wu L Yao J. Effects of Fulvic acid on performance of laying hens. Cereal Feed Industry. (2009) 9:36–7.

[19] Wang J Zhang H Wu S Yue H. Effects of dietary supplementation of fulvic acid on performance and blood biochemical indexes of broilers. Chinese Journal of Animal Nutrition (2013).

[20] Bi Y. Pharmacological experimental study of fulvic acid and sodium fulvic acid. Kunming University of Science and Technology (2009).

[21] Hudák M Semjon B Marcinčáková D Bujnák L Nad P Koréneková B. Effect of broilers chicken diet supplementation with natural and acidified humic substances on quality of produced breast meat. Animals. (2021) 11:1087. 10.3390/ani11041087 33920276 PMC8069141

[22] Domínguez-Negrete A Gómez-Rosales S Angeles MdL López-Hernández LH Reis de Souza TC Latorre-Cárdenas JD. Addition of different levels of humic substances extracted from worm compost in broiler feeds. Animals. (2021) 11:3199. 10.3390/ani11113199 34827930 PMC8614351

[23] Prokešová M Bušová M Zare M Tran HQ Kučerová E Ivanova AP. Effect of humic substances as feed additive on the growth performance, antioxidant status, and health condition of african catfish (Clarias gariepinus, Burchell 1822). Animals. (2021) 11:2266. 10.3390/ani11082266 34438724 PMC8388438

[24] Yörük M Gül M Hayirli A Macit M. The effects of supplementation of humate and probiotic on egg production and quality parameters during the late laying period in hens. Poult Sci. (2004) 83:84–8. 10.1093/ps/83.1.84 14761088

[25] Disetlhe ARP Marume U Mlambo V. Humic acid and enzymes inclusion in canola-based diets generate different responses in growth performance, protein utilization dynamics, and hemato-biochemical parameters in broiler chickens. Poult Sci. (2018) 97:2745–53. 10.3382/ps/pey047 29757447

[26] Hernandez F Garcia V Madrid J Orengo J Catalá P Megias M. Effect of formic acid on performance, digestibility, intestinal histomorphology and plasma metabolite levels of broiler chickens. Br Poult Sci. (2006) 47:50–6. 10.1080/00071660500475574 16546797

[27] Gutiérrez-Dagnino A Luna-González A Fierro-Coronado JA Álvarez-Ruíz P del Carmen Flores-Miranda M Miranda-Saucedo S. Effect of inulin and fulvic acid on survival, growth, immune system, and WSSV prevalence in Litopenaeus vannamei. Lat Am J Aquat Res. (2015) 43:912–21. 10.3856/vol43-issue5-fulltext-11

[28] Pan D Yu Z. Intestinal microbiome of poultry and its interaction with host and diet. Gut Microbes. (2014) 5:108–19. 10.4161/gmic.26945 24256702 PMC4049927

[29] Carding SR Davis N Hoyles L. Review article: the human intestinal virome in health and disease. Aliment Pharmacol Ther. (2017) 46:800–15. 10.1111/apt.14280 28869283 PMC5656937

[30] Sun B Wang X Bernstein S Huffman MA Xia DP Gu Z. Marked variation between winter and spring gut microbiota in free-ranging Tibetan Macaques (Macaca thibetana). Sci Rep. (2016) 6:26035. 10.1038/srep26035 27180722 PMC4867428

[31] Garneau JE Tremblay DM Moineau S. Characterization of 1706, a virulent phage from Lactococcus lactis with similarities to prophages from other Firmicutes. Virology. (2008) 373:298–309. 10.1016/j.virol.2007.12.002 18191977

[32] Miao V Davies J. Actinobacteria: the good, the bad, and the ugly. Antonie Van Leeuwenhoek. (2010) 98:143–50. 10.1007/s10482-010-9440-6 20390355

[33] Wang Y Zhang H Zhu L Xu Y Liu N Sun X. Dynamic distribution of gut microbiota in goats at different ages and health states. Front Microbiol. (2018) 9:2509. 10.3389/fmicb.2018.02509 30405569 PMC6207909

[34] Yang H Xiao Y Gui G Li J Wang J Li D. Microbial community and short-chain fatty acid profile in gastrointestinal tract of goose. Poult Sci. (2018) 97:1420–8. 10.3382/ps/pex438 29365165

[35] Kieler IN Shamzir Kamal S Vitger AD Nielsen DS Lauridsen C Bjornvad CR. Gut microbiota composition may relate to weight loss rate in obese pet dogs. Vet Med Sci. (2017) 3:252–62. 10.1002/vms3.80 29152318 PMC5677773

[36] Markovska R Stoeva T Dimitrova D Boyanova L Stankova P Mihova K. Quinolone resistance mechanisms among third-generation cephalosporin resistant isolates of Enterobacter spp. in a Bulgarian university hospital. Infect Drug Resist. (2019) 12:1445–55. 10.2147/IDR.S204199 31213860 PMC6549396

